# Aiding Grasp Synthesis for Novel Objects Using Heuristic-Based and Data-Driven Active Vision Methods

**DOI:** 10.3389/frobt.2021.696587

**Published:** 2021-07-15

**Authors:** Sabhari Natarajan, Galen Brown, Berk Calli

**Affiliations:** ^1^ Manipulation and Environmental Robotics Laboratory (MER Lab), Robotics Engineering Department, Worcester Polytechnic Institute, Worcester, MA, United States; ^2^ Manipulation and Environmental Robotics Laboratory (MER Lab), Computer Science Department, Worcester Polytechnic Institute, Worcester, MA, United States

**Keywords:** active vision, grasp synthesis, reinforcement learning, self-supervised learning, benchmarking

## Abstract

In this work, we present several heuristic-based and data-driven active vision strategies for viewpoint optimization of an arm-mounted depth camera to aid robotic grasping. These strategies aim to efficiently collect data to boost the performance of an underlying grasp synthesis algorithm. We created an open-source benchmarking platform in simulation (https://github.com/galenbr/2021ActiveVision), and provide an extensive study for assessing the performance of the proposed methods as well as comparing them against various baseline strategies. We also provide an experimental study with a real-world two finger parallel jaw gripper setup by utilizing an existing grasp planning benchmark in the literature. With these analyses, we were able to quantitatively demonstrate the versatility of heuristic methods that prioritize certain types of exploration, and qualitatively show their robustness to both novel objects and the transition from simulation to the real world. We identified scenarios in which our methods did not perform well and objectively difficult scenarios, and present a discussion on which avenues for future research show promise.

## 1 Introduction

Robotic grasping is a vital capability for many tasks, particularly in service robotics. Most grasping algorithms use data from a single viewpoint to synthesize a grasp (Caldera et al., 2018). This approach attempts to create a single, master algorithm that is useful for all objects in all situations. Nevertheless, these algorithms tend to suffer when the viewpoint of the vision sensor is different than the images used in training (Viereck et al., 2017). Additionally, many graspable objects have observation angles that are “singular” from which no grasp can be synthesized: For example, if an object has only one graspable surface, which is self-occluded from the current viewpoint of the camera, the grasp synthesis algorithm would either fail to find any grasps or would need to rely on assumptions that might not always hold, and therefore lead to an unsuccessful grasp attempt.

The issues of the single viewpoint approaches can be addressed *via* active vision frameworks, i.e., by actively moving the camera and collecting more data about the task. At one end of this spectrum is collecting data to obtain a complete 3D model of the object. This approach is slow, difficult to carry out in the real world, and vulnerable to misalignment if conditions change during or after data collection (Lakshminarayanan et al., 2017). Our aim is to develop active vision strategies that can efficiently collect data with brief motions and allow the grasp synthesis algorithms to find sufficiently good grasps as quickly as possible. It is shown in the grasping literature that even algorithms tailored for single viewpoints can have a substantial performance boost even with very simple data collection procedures (Viereck et al., 2017). Utilizing active vision for robotic grasping has several avenues for optimization: the exploration algorithm, the data analysis, and the grasping algorithm are all open questions.

**FIGURE 1:**
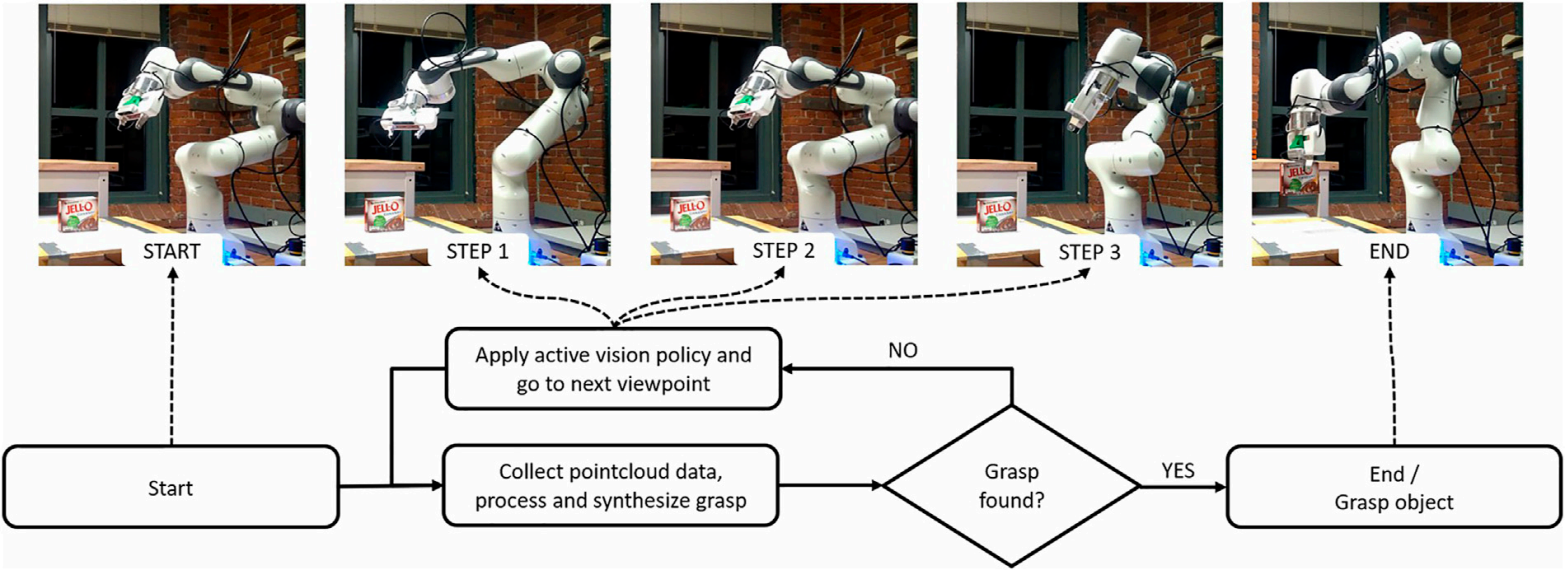
The 3D Heuristic policy guiding the camera and finding the grasp for a object.

In this work, we present a wide variety of exploration algorithms along with an extensive simulation and real-world experiment analysis. Figure 1 shows how an active vision policy explores different objects. In the simulation, we created benchmarks to assess not only whether our policies do better than random but to measure how close each approach comes to optimal behavior for each object. In the real-world experiments, we have adopted an existing grasp planning benchmark (Bekiroglu et al., 2020), and assess how well the simulation performances translate to real systems.

Our exploration algorithms can be split into heuristic and machine learning approaches. In our heuristics, we attempt to identify simple properties of the visual data that are reliable indicators of effective exploration directions. These approaches use estimates of how many potentially occluded grasps lie in each direction. For machine learning, we used self-supervised and Q-learning based approaches. We compare the performance of these methods against three baseline algorithms. The baselines are random motion (as the worst-case algorithm), naive straightforward motion (as a simple algorithm more complex efforts should outperform), and breadth-first-search (as the absolute ceiling on possible performance). The last is particularly important: because in the simulation we could exhaustively test each possible exploration path, we can say with certainty what the shortest possible exploration path that leads to a working grasp is. We also present a comparison study to another active vision-based algorithm, i.e., (Arruda et al., 2016), which provides, to the best of our knowledge, the closest strategy to ours in the literature.

To summarize, the contribution of our work is as follows:

1) We present two novel heuristic-based viewpoint optimization methods.
2) We provide a novel Q-learning based approach for achieving an exploration policy for grasping.
3) We provide an open-source simulation platform (https://github.com/galenbr/2021ActiveVision) to develop new active vision algorithms and benchmark them.
4) We present an extensive simulation and experimental analysis, assessing and comparing the performance of five active vision methods against three baseline strategies, including the optimal BFS strategy.

Taken together, these allow us to draw new conclusions not only about how well our algorithms work but how much it would be possible to improve them.

## 2 Related Works

Adapting robotic manipulation algorithms to work in an imperfect and uncertain world is a central concern of the robotics field, and an overview of modern approaches is given by Wang et al. (2020). At the same time, much of the research on robotic grasping does not attempt to move the vision sensor and focuses on single image grasp synthesis. Saxena et al. (2010)’s work on grasping objects in cluttered environments acknowledges the problem of accurately sensing a complex 3D environment, but attempts to avoid it by storing prebuilt 3D models and using them to better analyze a single stereovision image rather than by collecting more information. In a similar vein, Zheng et al. (2018) approaches industrial grasping by trying to more accurately map known features to objects instead of by trying to collect more data to resolve ambiguities in the images. A typical approach in literature is to train a neural network to produce grasps by annotating individual images with grasp candidates. This is the method used by Pinto and Gupta (2016), Chu et al. (2018), among many others. Even in tasks peripheral to grasping, like shape and pose estimation, considerable work has gone into more refined algorithms and machine learning strategies for extracting information from single 2D images without attempting to capture more images or optimize the viewpoint (Kurenkov et al., 2018; Zhang and Cao, 2019). Most work assumes that the viewpoint is fixed or random, and so focuses on either processing the data (pose estimation, object localization and segmentation, etc … ) or synthesizing a grasp from available data (Salganicoff et al., 1996; Du et al., 2021).

Our research focuses on this problem of collecting new data to improve processing outcomes. Active vision has been applied to many aspects of machine vision, but often with the explicit goal of completely viewing a 3D object (Khalfaoui et al., 2012; Daudelin and Campbell, 2017), rather than our objective of viewing just enough of the object to perform a grasp. Even in the narrower domain of active vision for grasp synthesis, not all work relates to our concerns. For instance Fu et al. (2019)’s study on industrial grasping uses active vision to assist feature identification of known objects, but with the explicit goal of maximizing grasp precision rather than minimizing information collected. For the use of active vision to address grasping using incomplete information, there has been research into both algorithmic (Calli et al., 2011; Arruda et al., 2016) and data-driven methods (Paletta and Pinz, 2000; Viereck et al., 2017; Calli et al., 2018b; Rasolzadeh et al., 2010), with more recent works tending to favor data-driven approaches (Caldera et al., 2018). In particular, the work in (Viereck et al., 2017) demonstrated that active vision algorithms have the potential to outperform state of the art single-shot grasping algorithms.

Calli et al. (2011) proposed an algorithmic active vision strategy for robotic grasping, extending 2D grasp stability metrics to 3D space. As an extension of that work (Calli et al., 2018b), the authors utilized local optimizers for systematic viewpoint optimization using 2D images. Arruda et al. (2016) employs a probabilistic algorithm whose core approach is the most similar to our heuristics presented in Section 4.2. Our approaches differ in focus, since Arruda et al. (2016) selects viewpoints based on estimated information gain as a proxy for finding successful grasps, while we prioritize grasp success likelihood and minimizing distance traveled. In our simulation study, we implemented a version of their algorithm and included it in our comparative analysis.

The data-driven approach presented in Viereck et al. (2017) avoided the problem of labeled data by automating data labeling using state of the art single shot grasp synthesis algorithms. They then used machine learning to estimate the direction of the nearest grasp along a view-sphere and performed gradient descent along the vector field of grasp directions. This has the advantage of being continuous and fast, but did not fit in our discrete testing framework (Viereck et al., 2017). All data-driven methods analyzed in this paper utilize a similar self-supervised learning framework due to its significant easiness in training.

One of our data-driven active vision algorithms utilizes the reinforcement learning framework. A similar strategy for active vision is used by Paletta and Pinz (2000) to estimate an information gain maximizing strategy for object recognition. We not only extend Q-learning to grasping, but do away with the intermediary information gain heuristic in reinforcement learning. Instead, we penalize our reinforcement approach for each step it takes that does not find a grasp, incentivizing short, efficient paths.

Two of the data-driven methods in this paper are based on the general strategy in our prior work in Calli et al. (2018a). In that work, we presented a preliminary study in simulation. In this paper, we present one additional variant of this strategy and present a more extended simulation analysis.

Gallos and Ferrie (2019), while focused on classification rather than grasping, heavily influenced our theoretical concerns and experimental design. Their paper argues that contemporary machine learning based active vision techniques outperform random searches but that this is too low a bar to call them useful and demonstrates that none of the methods they implemented could outperform the simple heuristic of choosing a direction and moving along it in large steps. Virtually all active vision literature [e.g., de Croon et al. (2009); Ammirato et al. (2017)] compares active vision approaches to random approaches or single-shot state of the art algorithms. While there has been research on optimality comparison in machine vision (Karasev et al., 2013), to the best of our knowledge, it has never been extended to 3D active vision, much less active vision for grasp synthesis. Our simulation benchmarks are an attempt to not only extend their approach to grasping, but to quantify how much improvement over the best performing algorithms remains possible.

## 3 Overview

**FIGURE 2:**
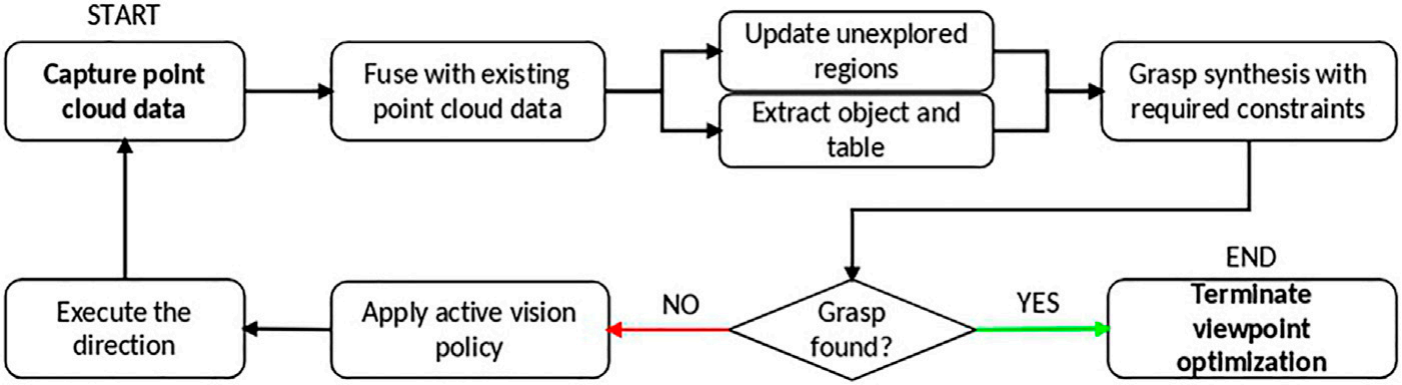
The active vision based grasp synthesis pipeline.

The proposed active vision based grasp synthesis pipeline is represented in Figure 2. It starts with collecting environment information from a viewpoint and fusing it with the previously known information about the environment (except for the first viewpoint captured). The object and table data are extracted, and the regions which have not been explored (unexplored regions) by the camera yet are updated. This processed data is used in the grasp synthesis and active vision policies as explained below. An attempt is made to synthesize a grasp with the available data, and if it fails, the active vision policy is called to guide the camera to a new viewpoint after which the process repeats until a grasp is found.

### 3.1 Workspace Description

**FIGURE 3:**
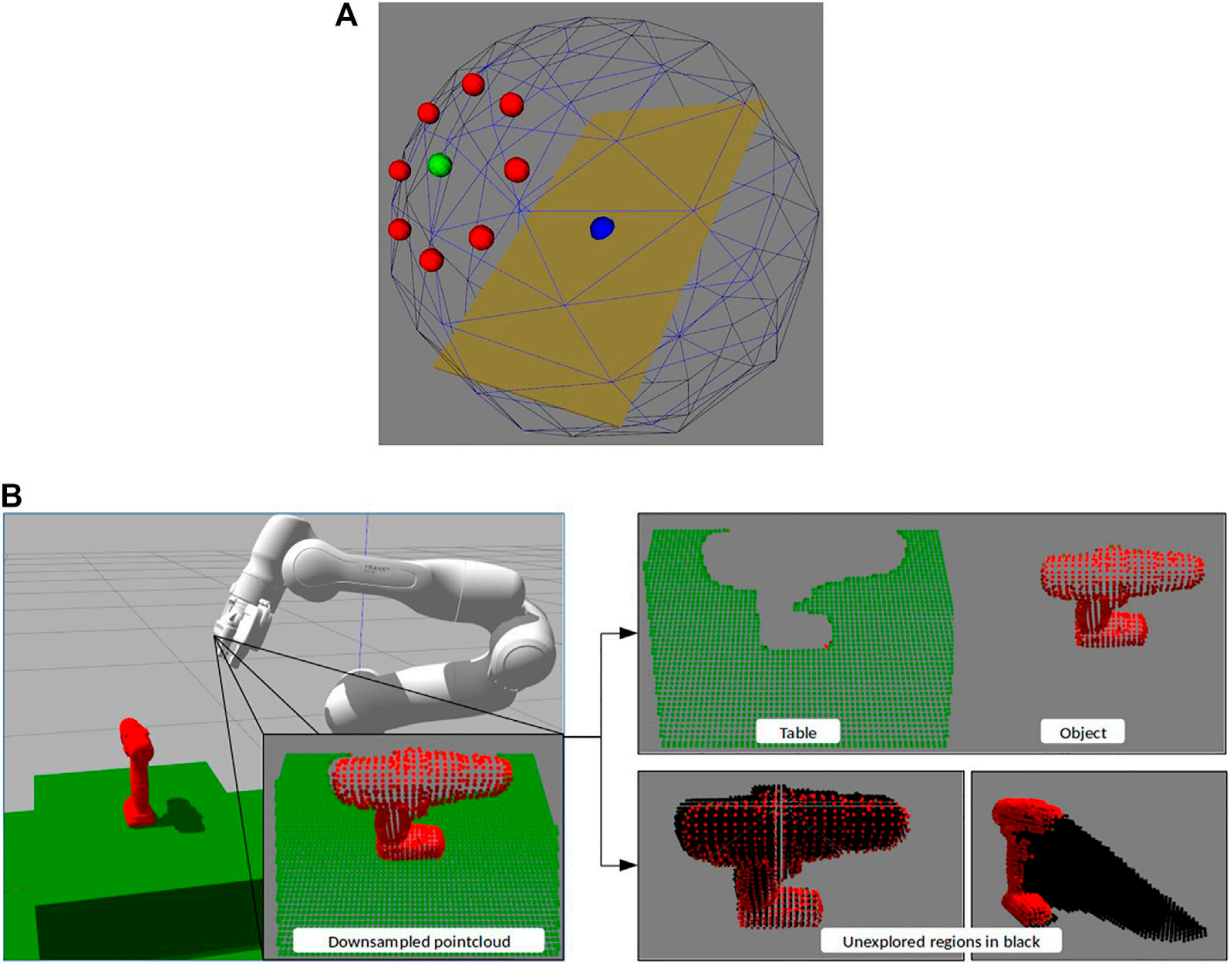
**(A)** Viewsphere and its next steps with parameters *v*_*r*_ = 0.4 m, *v*_*s*_ = 20°, and *v*_*d*_ = 8. The blue sphere is the expected position of the object, the green sphere the current camera position and red one the next steps it can take **(B)** Example with power drill as object showing the processed pointclouds. Left: Environment as seen by the camera, top-right: Extracted object and table, bottom-right: The unexplored regions of the environment.

We assume an eye-in-hand system that allows us to move the camera to any viewpoint within the manipulator workspace. To reduce the dimension of the active vision algorithm’s action space, the camera movement is constrained to move along a viewsphere, always pointing towards and centered around the target object [a common strategy also adopted in Paletta and Pinz (2000), Arruda et al. (2016), and Calli et al. (2018a)]. The radius of the viewsphere (*v*_*r*_) is set based on the manipulator workspace and sensor properties. In the viewsphere, movements are discretized into individual steps with two parameters, step-size (*v*_*s*_) and number of directions (*v*_*d*_). Figure 3A shows the workspace we use with *v*_*r*_ = 0.4 m, *v*_*s*_ = 20°, and *v*_*d*_ = 8 (N,NE,E,SE,S,SW,W,NW). In our implementation, we use an Intel Realsense D435i as the camera on the Franka Emika Panda arm for our eye-in-hand system.

### 3.2 Point Cloud Processing and Environment Modeling

The point cloud data received from the camera is downsampled before further processing to reduce sensor noise and to speed up the execution time. Figure 3B shows the environment as seen by the camera after downsampling.

Sample Consensus based plane segmentation techniques in Point Cloud Library (Rusu and Cousins, 2011) are used to extract the table information from the scene after which the points above the table are extracted and marked as object points. As mentioned previously, identifying the unexplored regions is required for grasp synthesis as well as the active vision policies. For this purpose, the region surrounding the object is populated with an evenly spaced point cloud and then sequentially checked to determine which points are occluded. While a common visibility check approach is ray-tracing, it is a computationally intensive and time consuming process. Instead, we take advantage of the organized nature of the point cloud data. The 3D points are projected to the image plane using:

$$X_{p}=KX/z_{0}$$ (1)

where, $X_{p}$ is the projected pixel co-ordinates, *X* is the 3D point ${\left(\begin{array}{c} x_{0}\;y_{0}\;z_{0} \end{array}\right)}^{T}$, and *K* is the camera intrinsic matrix described by:

$$K=\left(\begin{array}{ccc} f_{x} & 0 & pp_{x}\\ 0 & f_{y} & pp_{y}\\ 0 & 0 & 1 \end{array}\right)$$ (2)

The “depth value at $X_{p}$” and “$z_{0}$” are compared and if $z_{0}$ is greater than depth at $X_{p}$, the point *X* is marked as occluded. This approach reduces the computation time greatly. The two images on the bottom right of Figure 3B show the unexplored region generated for the drill object.

With every new viewpoint the camera is moved to, the newly acquired point cloud is fused with the existing environment data and the process is repeated to extract the object data and update the unexplored regions.

### 3.3 Grasp Synthesis

The goal of our pipeline is to provide enough data to an underlying grasp synthesis algorithm to find a successful grasp. As such, our methods are largely agnostic to the specific grasp synthesis algorithm used. Essentially, any gripper type i.e., parallel-jaw/multi-fingered/vacuum along with its corresponding grasp synthesis algorithm can be used in this methodology, provided the grasp synthesis algorithm can process an incomplete point cloud as input. However, these grasping algorithms are naturally preferred to be fast (since they will be run multiple times per grasp within our viewpoint optimization process), and be able to work with stitched point clouds. Most data-driven approaches in the literature are trained with single-view point clouds, and might not perform well with stitched object data.

In our study, we use the Franka Emika parallel jaw gripper (https://www.franka.de/) which has a maximum gripper width of 8 cm and a contact surface area of 4 cm^2^ as shown in Figure 3B. We use the term “contact point” to refer to a point in the point cloud being considered for a grasp. The point cloud library (PCL) (Rusu and Cousins, 2011) has modules for calculating the normal vector and curvature which are used in the following process. As our gripper is a parallel jaw gripper, we use a force-closure-based approach similar to Calli et al. (2018a), with the following constraints:

1) Grasp quality: This quality is a value ranging from 0 to 180 and depends on the normal vectors of the two contact points being considered for the grasp and their relative position. This is calculated as:

$$GQ=180-\left(min\left(∠\left(\vec{C_{1}C_{2}},\vec{C_{1N}}\right),∠\left(\vec{C_{2}C_{1}},\vec{C_{1N}}\right)\right)+min\left(∠\left(\vec{C_{1}C_{2}},\vec{C_{2N}}\right),∠\left(\vec{C_{2}C_{1}},\vec{C_{2N}}\right)\right)\right)$$ (3)

where, GQ is the grasp quality, $C_{1}$ and $C_{2}$ are the contact points 1 and 2 respectively, $C_{1N}$ and $C_{2N}$ are the surface normal vectors at the contact points 1 and 2 respectively. In this study a grasp quality between 150 and 180 was required.

2) Contact patch area and curvature constraint: To grasp the object, there must be a relatively flat surface surrounding each contact point at least as large as the gripper’s contact area. Based on the known gripper contact area and the points surrounding the contact point under consideration, the object contact patch area is calculated by projecting the points within a 3 cm radius of the contact point onto a plane whose normal vector is the same as the contact point’s. This projected area should be higher than a threshold for the contact point, and the curvature as calculated by PCL should be below a threshold.

The process of selecting a grasp i.e., a pair of contact points from the point cloud is described in Algorithm 1. Each grasp has infinitely many possible gripper orientations that would align with the contact points i.e., the gripper can revolve along the axis connecting the two points. The possible orientations have been discretized into eight evenly spaced orientations in this study. The collision and reachability checks (described in Algorithm 1) used to select a grasp from the possible grasps are computationally intensive and hence it is preferable to do as few checks as possible to select the grasp. Algorithm 2 explains the process of sorting the potential grasps using the grasp related parameters i.e., centroid, euclidean_distance, line_distance and grasp_quality. The thresholds used were experimentally determined. This sorting process helps to prioritize the grasps that have higher probability of passing the checks and which are closer to the centroid of the object. This reduces the number of checks required to arrive at the final grasp.

Algorithm 1: Grasp selection process.

**Require:** obj← Object point cloud
**Require:** tbl← Table point cloud
**Require:** unexp← Unexplored point cloud
 contact_pair← Pair of contact points
 potential_grasps← Array of contact_pair which can be a potential grasp
 **for all** contact_pair∈obj **do**
  **if** All constraints satisfied **then**
   contact_pair.euclidean_distance = euclidean distance between the contact_pair’s centroid and the obj’s centroid
   contact_pair.line_distance = euclidean distance between the contact_pair’s centroid and the obj’s line of gravity.
   Add contact_pair to potential_grasps
  **end if**
 **end for**
 Sort potential_grasps by Algorithm 2.
 **for all** contact_pair∈potential_grasps **do**
  **for** each of the eight gripper orientations **do**
   **if** No collision of gripper model with unexp and tbl and within manipulator workspace **then**
    Select this contact_pair at this gripper orientation
    Exit both the for loops
   **end if**
  **end for**
 **end for**.

Algorithm 2: Grasp sorting metric.

**Require:** potential_grasp_A← First contact_pair
**Require:** potential_grasp_B← Second contact_pair
 **if** abs(potential_grasp_A.line_distance - potential_grasp_B.line_distance) ≤.01 m **then**
  **if** abs(potential_grasp_A.centroid.z - potential_grasp_B.centroid.z) ≤.06 m **then**
   **if** abs(potential_grasp_A.euclidean_distance - potential_grasp_B.euclidean_distance) ≤.01 m **then**
    potential_grasp with the highest grasp_quality is preferred
   **else**
    potential_grasp with the lowest euclidean_distance is preferred
   **end if**
  **else**
   potential_grasp with the highest centroid.z is preferred
  **end if**
 **else**
  potential_grasp with the lowest line_distance is preferred
 **end if**.

Next, we explain the active vision policies designed and utilized in this paper.

## 4 Active Vision Policies

The focus of this paper is the active vision policies, which guide the eye-in-hand system to its next viewpoints. The nature of the pipeline allows us to plug in any policy which takes point clouds as its input and returns the direction to move for the next viewpoint. The policies developed and tested in this paper have been classified into three categories as follows:

1) Baseline policies.
2) Heuristic policies.
3) Machine learning policies.

Each of these sets of policies are explained below.

### 4.1 Baseline Policies

As the name suggests these are a set of policies defined to serve as a baseline to compare the heuristic and machine learning policies with. The three baselines used are shown below.

#### 4.1.1 Random Policy

Ignoring camera data, a random direction was selected for each step. No constraints were placed on the direction chosen, leaving the algorithm free to (for instance) oscillate infinitely between the start pose and positions one step away. This represents the worst case for an algorithm not deliberately designed to perform poorly, and all methods should be expected to perform better than it in the aggregate. This is the standard baseline in the active vision literature.

#### 4.1.2 Brick Policy

Named after throwing a brick on the gas pedal of a car, a consistent direction (North East) was selected at each timestep. This direction was selected because early testing strongly favored it, but we make no claims that it is ideal. This policy represents the baseline algorithm that is naively designed and which any serious algorithm should be expected to outperform, but which is nonetheless effective. Any algorithm that performed more poorly than it would need well-justified situational advantages to be usable.

#### 4.1.3 Breadth-First-Search Policy

From the starting position, an exhaustive Breadth-First-Search was performed, and an optimal path selected. This policy represents optimal performance, as it is mathematically impossible for a discrete algorithm to produce a shorter path from the same start point. No discrete method can exceed its performance, but measuring how close each method comes to it gives us an objective measure of each method’s quality in each situation.

With baselines defined, we will now discuss the other categories starting with heuristics.

### 4.2 Heuristic Policies

The idea behind the heuristic policy is to choose the best possible direction after considering the next available viewpoints. The metric used to define the quality of each of the next viewpoints is a value proportional to the unexplored region visible from a given viewpoint.

#### 4.2.1 2D Heuristic Policy

**FIGURE 4:**
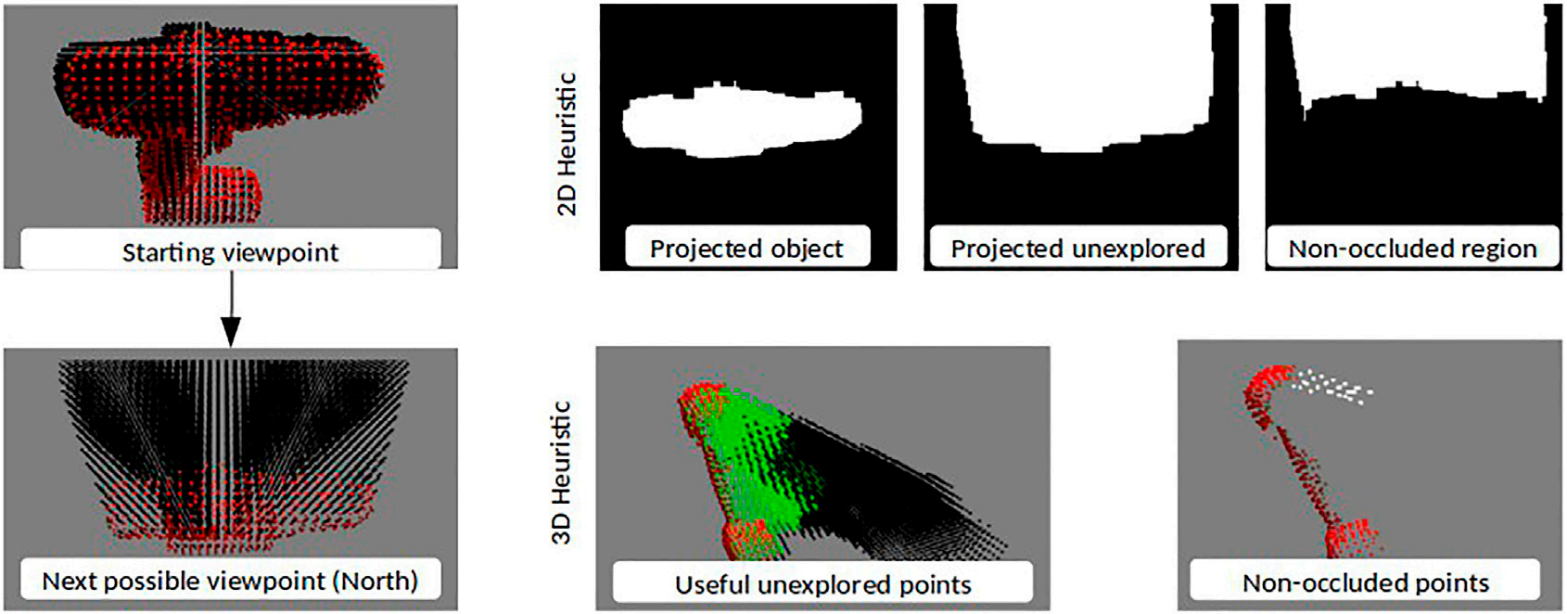
Set of images illustrating how the 2D and 3D Heuristics evaluate a proposed next step North with the drill object. The 3D Heuristic images have been shown from a different viewpoint for representation purposes.

The viewpoint quality is calculated by transforming the point clouds to the next possible viewpoints, and projecting the object and unexplored point clouds from those viewpoints onto an image plane using the camera’s projection matrix. This process has the effect of making the most optimistic estimation for exploring unexplored regions; it assumes no new object points will be discovered from the new viewpoint. Since the point clouds were downsampled, their projected images were dilated to generate closed surfaces. The 2D projections are then overlapped to calculate the size of the area not occluded by the object. The direction for which the most area of the unexplored region is revealed is then selected. Figure 4 shows an illustration with the dilated projected surfaces and the calculated non-occluded region. The 2D Heuristic policy is outlined in Algorithm 3.

Algorithm 3: 2D Heuristic policy.

**Require:** obj← Object point cloud
**Require:** unexp← Unexplored point cloud
 **for all** viewpoint∈ next possible viewpoints **do**
  **if** viewpoint within manipulator workspace **then**
   obj_trf← Transform obj to viewpoint
   obj_proj← Project obj_trf onto image plane (B/W image) and dilate
   unexp_trf← Transform unexp to viewpoint
   unexp_proj← Project unexp_trf onto image plane (B/W image) and dilate
   non_occ_unexp_proj←unexp_proj−obj_proj
  **end if**
  Record the number of white pixels in non_occ_unexp_proj
 **end for**
 Choose the direction with maximum white pixels.

While this heuristic is computationally efficient, it considers the 2D projected area, leading it to, at times, prefer wafer-thin slivers with high projected area over deep blocks with low projected area. Additionally, it is agnostic to the grasping goal and only focuses on maximizing the exploration of unseen regions.

#### 4.2.2 3D Heuristic Policy

In the 3D heuristic, we focused only on the unexplored region which could lead to a potential grasp. This was done using the normal vectors of the currently visible object. Since our grasp algorithm relies on antipodal grasps, only points along the surface normals can produce grasps. These points were extracted by using a 3D bounding box with a length of twice the maximum gripper width [2*8 cm], and a width and height of 1 cm. The longer axis of this box was aligned with the normal vector and the center of the box was aligned with the point in consideration. This was done for all object points to create a 3D mask of all unexplored space that could contain a grasp. The unexplored points which were outside this mask were discarded for the next steps.

Next, like in the 2D heuristic, we transformed the points to the next possible viewpoints. This time, instead of projecting, we used local surface reconstruction and ray-tracing to determine all the unexplored points which will not be occluded from a given viewpoint. The direction which leads to the highest number of non-occluded unexplored points is selected. This prioritizes exploring the greatest possible region of unexplored space that, based on known information, could potentially contain a grasp. If all the viewpoints after one step have very few non-occluded points the policy looks one step ahead in the same direction for each before making the decision. Figure 4 shows an illustration with the non-occluded useful unexplored region. The green points are the region of the unexplored region which is considered useful based on gripper configuration. The 3D Heuristic policy is outlined in Algorithm 3.

Algorithm 4: 3D Heuristic policy.

**Require:** obj← Object point cloud
**Require:** unexp← Unexplored point cloud
**Require:** points_threshold← Minimum number of non-occluded unexplored points needed for a new viewpoint to be considered useful
 useful_unexp Unexplored points with potential for a successful grasp
 **for all** viewpoint∈ next possible viewpoints **do**
  **if** viewpoint within manipulator workspace **then**
   obj_trf← Transform obj to viewpoint
   useful_unexp_trf← Transform useful_unexp to viewpoint
   non_occ_useful_unexp← Check occlusion for each useful_unexp_trf using local surface reconstruction and ray-tracing.
  **end if**
  Record the number of points in non_occ_useful_unexp
 **end for**
 max_points← Maximum points seen across the possible viewpoints
 **if** max_points≤points_threshold **then**
  Run the previous for loop with twice the step-size
 **end if**
 max_points← Maximum points seen across the possible viewpoints
 Choose the direction which has max_points

#### 4.2.3 Information Gain Heuristic Policy

The closest approach to the heuristics presented in this paper is provided by Arruda et al. (2016). For comparison purposes, we implemented an approximate version of their exploration policy to test our assumptions and compare it with our 3D Heuristic approach. First, we defined a set of 34 viewpoints across the viewsphere to replicate Arruda et al. (2016)’s search space. The same viewsphere setup as seen in Figure 3A was used. Each viewpoint is defined by a pair of polar and azimuthal angles. Three polar angle values of 22.5°, 45°, and 67.5° were used with 10, 12, and 12 evenly distributed azimuthal angle values from 0° to 360° respectively. To calculate the information gain for each viewpoint, we modified the 3D Heuristic to consider all unexplored regions as opposed to focusing on the regions with a potential grasp. Similarly, the modified 3D Heuristic policy, instead of comparing the next $v_{d}$ viewpoints, compared all 34 viewpoints and used the one with the highest information gain. A simulation study was performed to compare the camera travel distance and computation times of this algorithm to our other heuristics.

### 4.3 Machine Learning Policies

**FIGURE 5:**
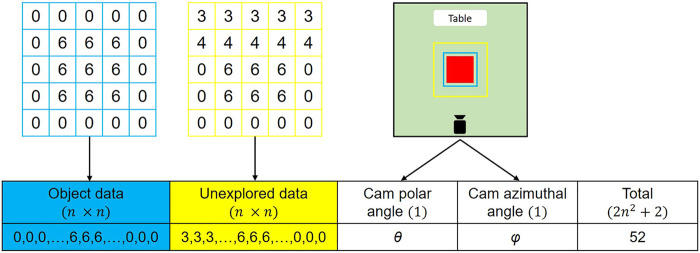
HAF based state vector for a 6 × 6 × 6 cm cube on the table. The blue square shows the square zone considered for object data and the yellow square shows the zone considered for unexplored data. The length of the unexplored data square zone is 1.5 times that of the object square zone. The values represent the maximum height among the points within each grid. These data are flattened and merged along with camera information for generate the state vector.

Our data-driven policies utilize a fixed-size state vector as input. This state vector is obtained by modeling the object point cloud and unexplored regions point cloud with Height Accumulated Features (HAF), developed by Fischinger and Vincze (2012) and used in Calli et al. (2018a) along with the camera position. We experimented with grid sizes of 5 and 7 height maps, both of which provide similar performance in our implementation, so we chose to use 5 for performance reasons. The state vector of a given view is composed of the flattened height maps of the extracted object and the unexplored point cloud and the polar and azimuthal angle of the camera in the viewsphere. The size of the state vector is $2n^{2}+2$, where *n* is the grid size. Figure 5 shows an illustration of the HAF state vector generation process.

#### 4.3.1 Self-Supervised Learning Policy

**FIGURE 6:**
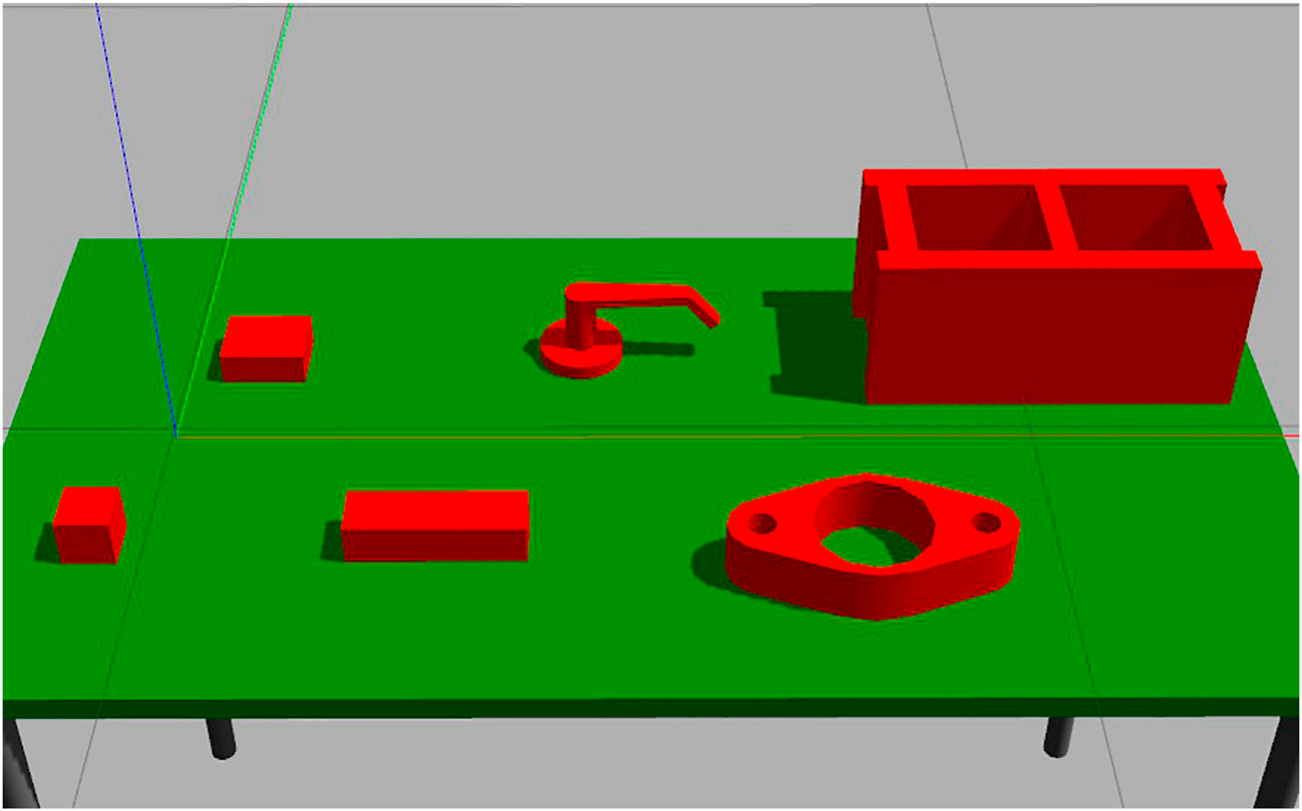
The set of objects used for simulation training. Filenames left to right: prism 6 × 6 × 6, prism 10 × 8 × 4, prism 20 × 6 × 5, handle, gasket, cinder block. All dimensions are in centimeters.

**FIGURE 7:**
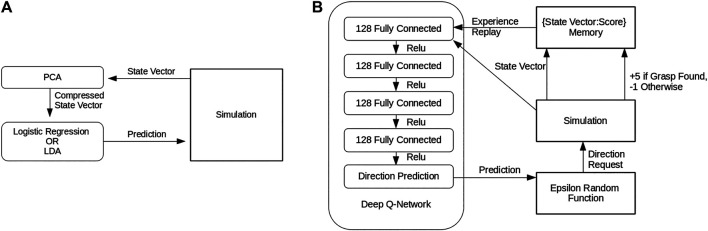
**(A)** The Self-supervised learning policy pipelines. Note that both methods are identical except for the specific self-supervised learning method used to process the compressed state vector **(B)** The Deep Q-Learning policy pipeline.

Following the synthetic data generation procedure used by Calli et al. (2018a), we generated training data in simulation. For a given start pose, each compass direction (North, North-East, East, etc … ) was explored for a grasp. If none were found, a further exploration of four random steps from each compass direction was performed three times. The shortest working path was saved, along with the state vector of each camera view in the path. This was repeated for 1,000 random initial poses each for the 10 × 8 × 4 and 20 × 6 × 5 prisms in Figure 6. Further training objects were considered, but initial results generalized so well that it was not pursued. This data was used to train two self-supervised learning methods, logistic regression and LDA classification, to predict the next best viewpoint to select given the state vector of the current viewpoint. In both of the methods, we first applied PCA to each state vector to further compress it to 26 components, as shown in Figure 7A. All the components used in this policy were implemented using the scikit-learn library (Pedregosa et al., 2011).

#### 4.3.2 Deep Q-Learning Policy

In an attempt to improve on the self-supervised methods, a deep Q-Learning policy was also trained to predict, for a given state vector, the next best viewpoint using Keras library tools (Chollet, 2015). Four fully connected 128 dense layers and one 8 dense layer, connected by Relu transitions, formed the deep network that made the predictions as shown in Figure 7B. In training, an epsilon-random gate replaced the network’s prediction with a random direction if a random value exceeded an epsilon value that decreased with training. The movement this function requested was then performed in simulation, and the resulting state vector and a binary grasp found metric were recorded. Once enough states had been captured, experience replay randomly selected from the record to train the Q-Network on a full batch of states each iteration. The Q-Learning was trained in simulation to convergence on all of the objects in Figure 6, taking roughly 1,300 simulated episodes to reach convergence. We hoped that, given the relatively constrained state space and strong similarities between states, meaningful generalizations could be drawn from the training set to completely novel objects.

For all machine learning approaches, the objects used for training were never used in testing.

## 5 Simulation and Experimental Results

**FIGURE 8:**
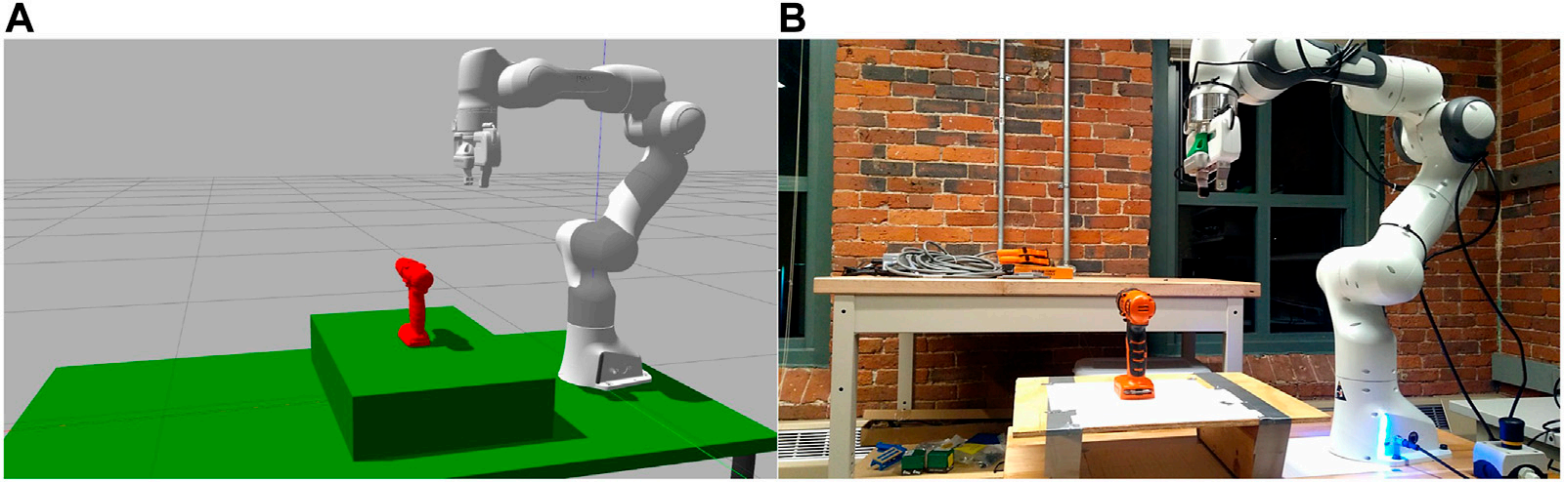
The setup as seen in simulation environment **(A)** and lab environment **(B)** with the power drill object in place.

The methodology discussed in the above section was implemented and tested in both simulation and in the real world. The setups used for the testing are shown in Figure 8. The maximum number of steps allowed before an experiment is restarted was set to six on the basis of preliminary experiments with the BFS policy.

### 5.1 Simulation Study

**FIGURE 9:**
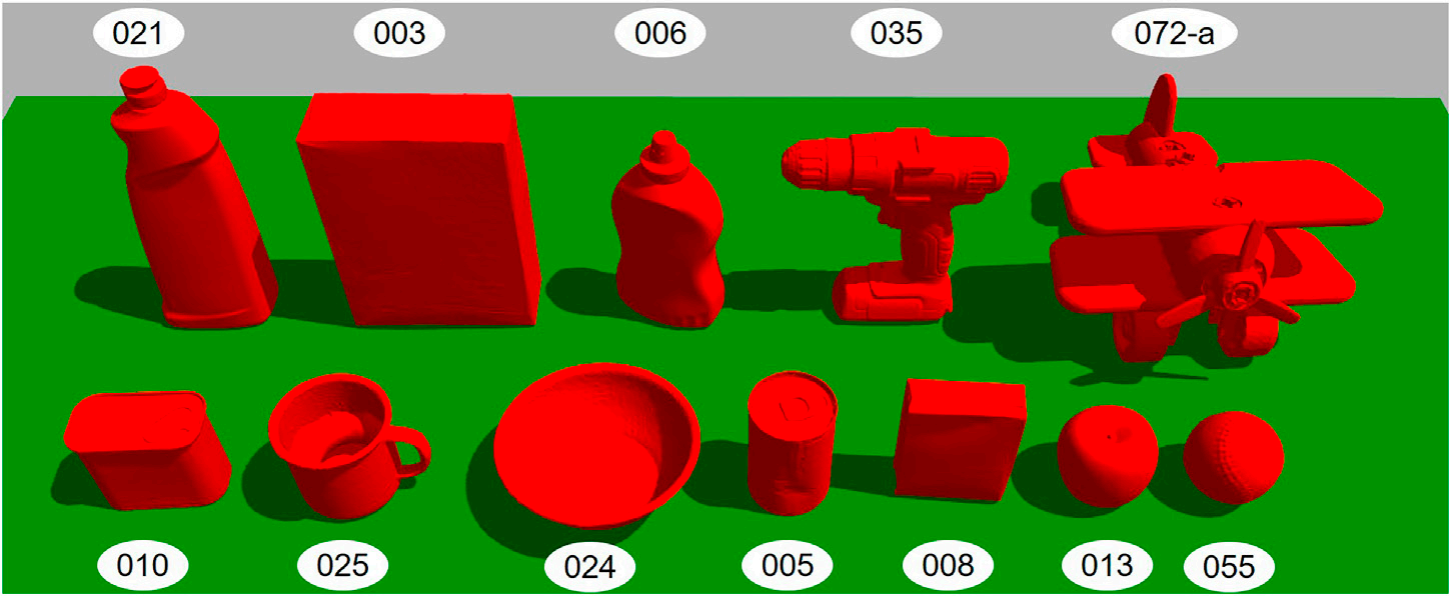
The set of objects used for simulation testing. Each object is labeled with its YCB ID.

The extensive testing in the simulation was done on a set of 12 objects from the YCB dataset (Calli et al., 2015) which are shown in Figure 9. To ensure consistency, we applied each algorithm to the exact same 100 poses for each object. This allowed us to produce a representative sample of a large number of points without biasing the dataset by using regular increments, while still giving each algorithm exactly identical conditions to work in. This was done by generating a set of 100 random values between 0 and 359 before testing began. To test a given policy with a given object, the object was spawned in Gazebo in a stable pose, with 0° of rotation about the *z*-axis. The object was then rotated by the first of the random value about the *z*-axis, and the policy was used to search for a viable grasp. After the policy terminated, the object was reset, and rotated to the second random value, and so on.

**FIGURE 10:**
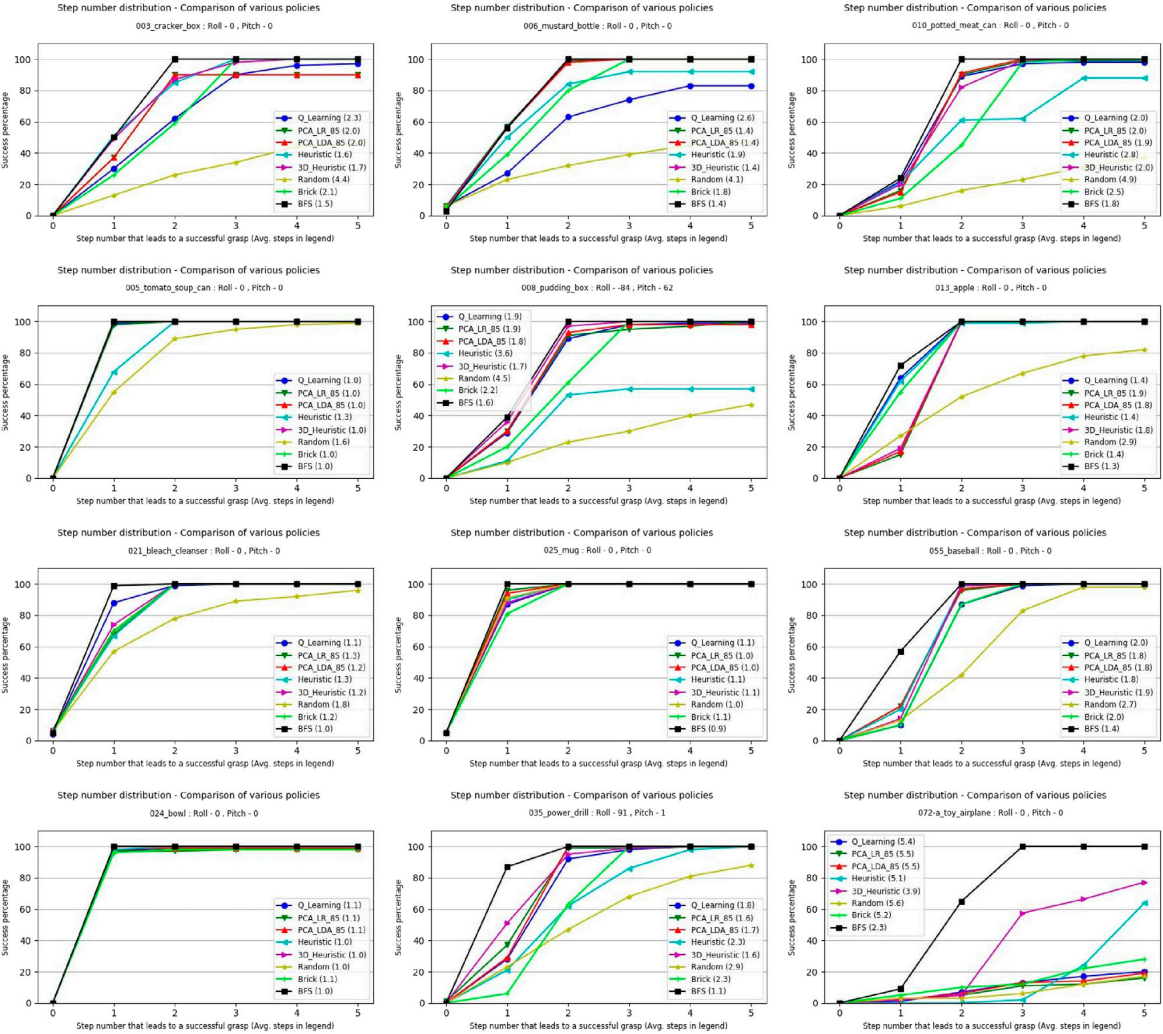
Simulation results for applying each approach to each object in 100 pre-set poses. Success is defined as reaching a view containing a grasp above a user defined threshold. The number in parenthesis by the policy names in the legend is the average number of steps that policy took to find a grasp. For cases where no grasp was found, the step count was considered to be 6.

The number of steps required to synthesize a grasp was recorded for each of the objects in each of its 100 tested poses. The success rate after each step for each object and the policies tested is shown in Figure 10. Each sub-image displays the fraction of poses a successful grasp has been reached for each policy on the same 100 pre-set poses for the given object. In object 025, for instance, the BFS found a working grasp on the first step for every starting pose, while all the other methods only found a grasp in the first step for a large majority of poses. By the second step, every policy has found a working grasp for every tested pose of object 025.

The use of baseline policies i.e., random for the lower limit and BFS for the upper limit allowed us to classify the objects as easy, medium, and hard in terms of how difficult is it to find a path that leads to a successful grasp. Objects are “Easy” when taking a step in almost any direction will lead to a successful grasp, and “Hard” when a low ratio of random to BFS searches succeed, suggesting very specific paths are needed to find a grasp. Two objects with similar optimal and random performance will have similar numbers of paths leading to successful grasps, and so differences in performance between the two would be due to algorithmic differences, not inherent difficulty. The random to BFS ratio is used for the classification. For example, if the BFS result shows that out of 100 poses 40 poses have a successful grasp found in the first step and a policy is only able to find a grasp at the first step for 10 poses, the policy is considered to have performed at 25% of the optimal performance or in other words the ratio would be 0.25. Objects with random to BFS ratio at Step 2 ≤ 0.40 are considered hard, objects between 0.41 and 0.80 as medium, and objects with a ratio > 0.80 as easy. With this criteria the test objects were classified as follows:

1) Easy: Tomato soup can (005), Bowl (024), Mug (025).
2) Medium: Apple (013), Bleach cleanser (021), Power drill (035), Baseball (055).
3) Hard: Cracker box (003), Mustard Bottle (006), Pudding box (008), Potted meat can (010), Toy airplane (072-a).

**FIGURE 11:**
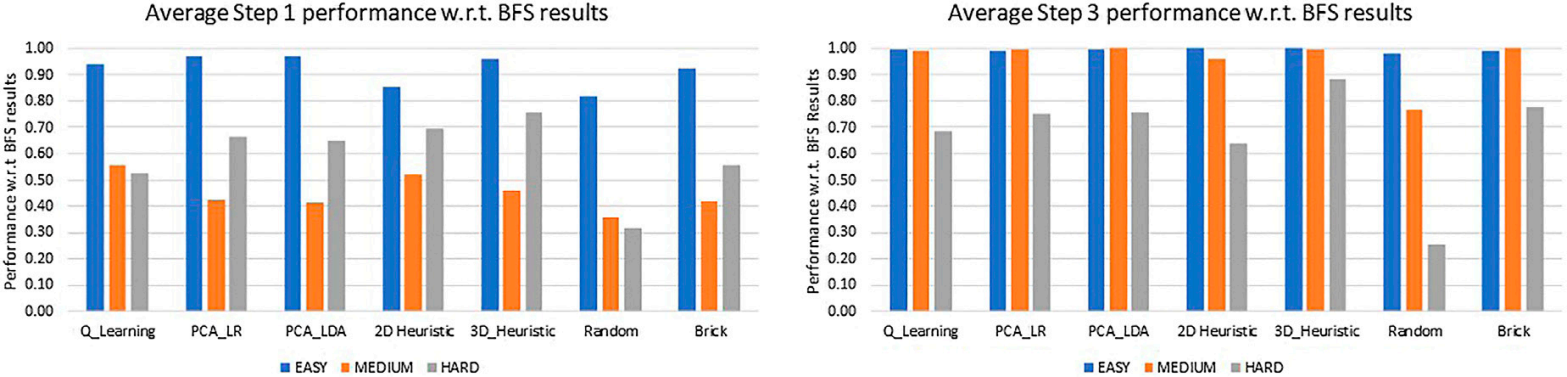
A comparison of performance of various policies for objects categorized into easy, medium and hard, for Step 1 and Step 3 with respect to the BFS performance at those steps.

With these object classifications, Figure 11 shows the performance of the policies for Step 1 and Step 3 using the policy to BFS ratio.

Figures 10, 11 show that overall in simulation, the 3D Heuristic performed the best, followed by the self-supervised learning approaches, Q-Learning and the 2D Heuristic. For half of the objects we tested, the 3D Heuristic performed best, while for objects 003, 010, 013, 021, 025, and 055 another algorithm performed better.

One reason the 3D Heuristic may be failing in some cases is that the heuristics are constrained to only considering the immediate next step. Our machine learning approaches can learn to make assumptions about several steps in the future, and so may be at an advantage on certain objects with complex paths. In addition, the optimistic estimations explained in Section 4.2.2 will not hold for all objects and cases, causing the Heuristic to waste time exploring promising looking dead-ends. One reason the machine learning techniques underperform for some cases may be due to the HAF representation used to compress the point cloud data, which creates a very coarse-grained representation of the objects, obliterating fine details. Additionally, HAF representations cannot represent certain types of concavities, hampering their utility for complex objects. A much finer grid size, or an alternative representation of the state vector, could help in improving the performance of the machine learning techniques.

We found that all methods consistently outperformed random, even on objects classified as hard. It is important to note that even brick policy was able to find successful grasps for all objects except for the toy airplane object (072-a), suggesting that incorporating active vision strategies even at a very basic level can improve the grasp synthesis for an object.

The toy airplane object (072-a) deserves special attention as it was by far the hardest object in our test set. It was the only object tested for which most algorithms did not achieve at least 80% optimal performance by step 5, as well as having the lowest random to BFS ratio at step 5. We also saw (both here and in the real world experiments) that heuristic approaches performed the best on this complex and unusual object, while the machine learning based approaches all struggled to generalize to fit it.

Easy and Medium category objects come very close to optimal performance around step 3, as seen in Figure 11. Given how small the possible gains on these simple objects can be, difficult objects should be the focus of future research.

Of the methods we examined, Heuristics (2D, 3D, and information gain) had the advantage of fast set up time, since they did not need training, but longer run time, since they performed more complicated calculations. The deep Q-learning had the disadvantage of needing extensive training time, but ran quickly, and the self-supervised learning approaches (LDA and logistic regression) could be trained quickly and ran quickly, but needed a long initial data collection period.

### 5.2 Comparison With the Information Gain Heuristic

Using the same simulation setup the Information Gain Heuristic policy [our implementation of Arruda et al. (2016)] was compared to the 3D heuristic policy. The comparison results are shown in Table 1, where the number of viewpoints required was converted to the effective number of steps for the 3D Heuristic for comparison. One step is the distance traveled to move to an adjacent viewpoint along the viewsphere in the discretized space with *v*_*r*_ = 0.4 m, *v*_*s*_ = 20.

**TABLE 1:** Comparison between the exploration pattern employed by the Information Gain Heuristic and the 3D Heuristic’s grasp weighted exploration.

Object name	Steps comparison				Timing comparison (s)		
	Information Gain Heuristic		3D Heuristic	Reduction %	Information Gain Heuristic	3D Heuristic	Reduction %
	nVPs	nEffSteps	nSteps				
Tomato soup can (005)	1	2.3	1	57	22	3	86
Mustard bottle (006)	0.92	2.1	1.4	33	35	10	71
Pudding box (008)	1	2.8	1.6	43	26	5	81
Potted meat can (010)	1.2	3.3	2	39	22	5.5	75
Apple (013)	1	2.3	2	13	9	4	56
Bowl (024)	1	2.9	1	66	14	3	79
Mug (025)	1	2.3	1	57	17	3	82
Power drill (035)	1.2	2.9	2.2	24	54	19	65
Toy airplane (072-a)	1.6	4.7	3	36	75	28	63
**Average**				41	**Average**		73

nVPs, Number of viewpoint visited apart from the starting viewpoint to find a grasp; nEffSteps, Distance traveled between viewpoints converted to number of “3D Heuristic” steps; nSteps, Number of steps taken by 3D Heuristic policy to find a grasp.

We see an average of 41% reduction in camera movement and with the 3D Heuristic policy, confirming our theory that only certain types of information warrant exploration and that by focusing on grasp containing regions we can achieve good grasps with much less exploration. As a side benefit, we also see a 73% reduction in processing time with the 3D Heuristic policy, as it considers far fewer views in each step.

### 5.3 Real World Study

**FIGURE 12:**
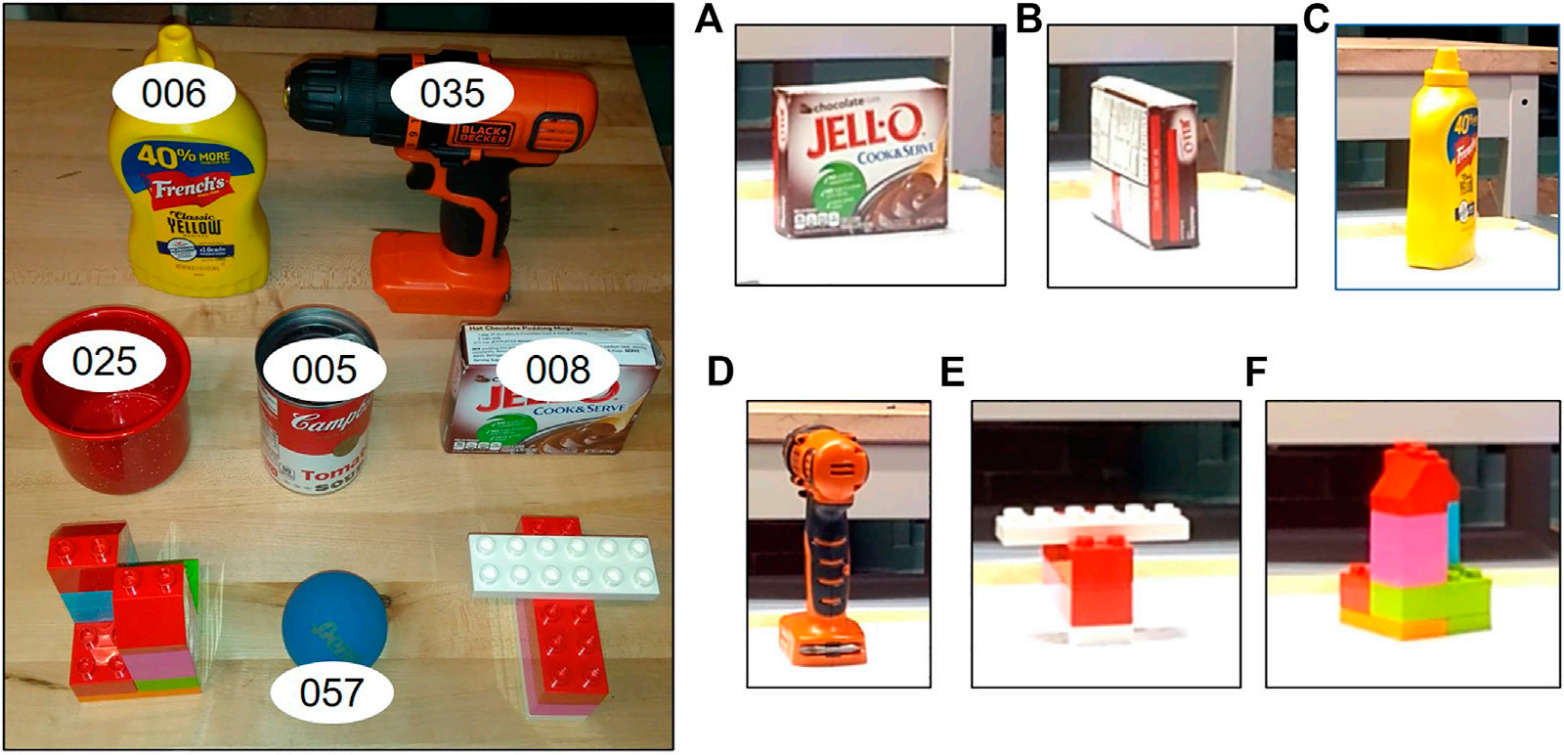
The left image shows the set of objects used for real world testing along with their YCB IDs. On the right are the stable poses used for testing (The manipulator base is towards the right). **(A)** [YCB ID : 008] Stable Pose \#1, **(B)** [YCB ID : 008] Stable Pose \#2, **(C)** [YCB ID : 006] Stable Pose \#1, **(D)** [YCB ID : 035] Stable Pose \#1, **(E)** [Custom Lego 1] Stable Pose \#1, **(F)** [Custom Lego 2] Stable Pose \#1. Objects with YCB IDs 005 and 057 are considered symmetrical and are used in the same orientation as shown in left image. Likewise, object 025 was tested in only one stable pose, with the handle facing away from the robot.

The real world testing was done on a subset of objects in simulation along with two custom objects built using lego pieces. The grasp benchmarking protocol in (Bekiroglu et al., 2020) was implemented to assess the grasp quality based on the five scoring parameters specified. Another grasp benchmarking protocol focused on vision-based approaches is Kootstra et al. (2012), but it is simulation based and needs the input to be in the form of stereo images which does not fit well with our pipeline’s need for a point cloud input. Also, it lacks the shaking and rotational test metrics available in Bekiroglu et al. (2020). The 3D Heuristic and the Q-Learning policies were selected and tested with the objects. The results for the tests performed are shown in Table 2. A total of 18 object-pose-policy combinations were tested with three trials for each and the average across the trails has been reported. The objects used along with their stable poses used for testing are shown in Figure 12.

**TABLE 2:** A list of objects tested for 3D Heuristic and QLearning policies along with the benchmarking results.

Object name	Stable pose	Policy	Policy success percentage	Avg steps taken	Avg grasp quality	Benchmarking results				
						C1	C2	C3	C4	C5
Chocolate Pudding box (008)	1	Q Learning	67	5.50	167	434	8	100	100	100
		3D Heuristic	100	3.00	167	205	4	100	100	100
	2	Q Learning	100	3.00	172	680	9	100	100	100
		3D Heuristic	100	3.00	171	675	12	100	100	100
Mustard bottle (006)	1	Q Learning	100	3.00	158	430	15	33 (F2)	0 (F3)	–
		3D Heuristic	100	2.33	161	370	6	33 (F2)	0 (F3)	–
Metal Mug (025)	1	Q Learning	100	2.00	180	125	40	100	100	100
		3D Heuristic	100	2.00	180	80	20	100	100	100
Tomato soup can (005)	1	Q Learning	0	–	–	–	–	–	–	–
		3D Heuristic	100	3.00	166	331	2	100	100	100
Racquet ball (057)	1	Q Learning	100	4.00	150	40	2	100	100	100
		3D Heuristic	100	5.00	154	32	2	100	100	100
Power drill (035)	1	Q Learning	100	3.00	164	351	1	100	100	100
		3D Heuristic	100	2.00	165	450	3	100	100	100
Custom lego object 1	1	Q Learning	0	–	–	–	–	–	–	–
		3D Heuristic	100	3.00	162	230	4	100	100	100
Custom lego object 2	1	Q Learning	0	–	–	–	–	–	–	–
		3D Heuristic	100	4.50	168	180	1	100	100	100

**FIGURE 13:**
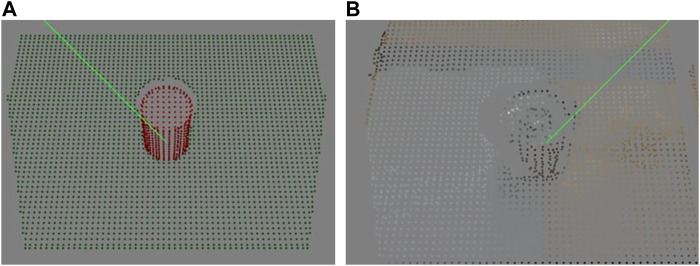
Difference between information captured by depth sensor in simulation **(A)** and real world **(B)** for the tomato soup can (005) object. Notice the lesser surface information captured by the real world sensor.

In real world trials, we found that the 3D heuristic works consistently, but the Q-Learning is at times unreliably. When run in simulation, the paths Q-Learning picks for the real-world objects produce successful grasps - the difference between our depth sensor in simulation and the depth sensor in the real world seems to be causing the disconnect. Figure 13 shows the difference between the depth sensors in the two environments for the tomato soup can (005) object. Apart from the noise which is present in real world sensor, the real sensor sees less surface information than its simulation counterpart. Notably, the surfaces that are more or less perpendicular to the sensor image plane are not seen in the real world sensor, but can be seen by the sensor in simulation. This explains why more steps were required in the real world than in simulation. Nonetheless, the reliability of the 3D Heuristic demonstrates that simulated results can be representative of reality, although there are some differences.

## 6 Conclusion

In this paper, we presented heuristic and data-driven policies to achieve viewpoint optimization to aid robotic grasping. In our simulation and real world testing, we implemented a wide variety of active vision approaches and demonstrated that, in overall performance, the 3D Heuristic outperformed both data-driven approaches and naive algorithms. We concluded that prioritizing exploration of grasp-related locations can produce both faster and more accurate heuristic policies. Also, we noticed that the data-driven policies had an edge over heuristic polices for some objects due to its inherent nature of considering multiple steps ahead as opposed to the heuristic policies which can see only one step ahead. From our optimal search, we demonstrated that for most objects tested, both types of approaches perform close to optimal. We were able to identify that the complex objects in our test set like the toy airplane and custom Lego objects are not only dissimilar to our training objects, but they are also objectively more difficult for viewpoint optimization. In the real world testing, we demonstrated that while sensor differences impacted all algorithms’ performances, the heuristic based approach was sufficiently robust to generalize well to the real world while our data-driven approaches were more sensitive to the changes in sensor behavior.

Both types of policies, i.e., heuristic-based and data-driven, had their pros and cons. The execution times of the policies were less than 1 s for the data-driven policies and for the heuristic ones they ranged from 0.5 to 5 s based on the size of the target object. The speed difference is due to the processing of raw point cloud data in the heuristic policies as opposed to the compressed state vector used in the data-driven policies. This data compression removes potentially useful information. The nature of our state-vector makes data-driven policies less reliable as seen with the tests involving the custom Lego objects. Using different data compression methods to generate the state vectors containing more data could be used to enhance the performance of the data-driven techniques along with using more objects for training and testing.

Future research should prioritize what we have identified as difficult objects over simple ones, as it is only in the more difficult objects that gains can be made and good policies discerned from poor ones. Additionally, our work depended on discrete movements through the viewsphere. Future work should consider the possibility that continuous motion through the viewsphere may outperform discrete strategies.

## Data Availability

The raw data supporting the conclusions of this article will be made available by the authors, without undue reservation.
